# Mechanisms Mediating the Effects of **γ**-Tocotrienol When Used in Combination with PPAR**γ** Agonists or Antagonists on MCF-7 and MDA-MB-231 Breast Cancer Cells

**DOI:** 10.1155/2013/101705

**Published:** 2013-01-28

**Authors:** Abhita Malaviya, Paul W. Sylvester

**Affiliations:** College of Pharmacy, University of Louisiana at Monroe, 700 University Avenue, Monroe, LA 71209, USA

## Abstract

**γ**-Tocotrienol is a natural vitamin E that displays potent anticancer activity, and previous studies suggest that these effects involve alterations in PPAR**γ** activity. Treatment with 0.5–6 **μ**M  **γ**-tocotrienol, 0.4–50 **μ**M PPAR**γ** agonists (rosiglitazone or troglitazone), or 0.4–25 **μ**M PPAR**γ** antagonists (GW9662 or T0070907) alone resulted in a dose-responsive inhibition of MCF-7 and MDA-MB-231 breast cancer proliferation. However, combined treatment of 1–4 **μ**M  **γ**-tocotrienol with PPAR**γ** agonists reversed the growth inhibitory effects of **γ**-tocotrienol, whereas combined treatment of 1–4 **μ**M  **γ**-tocotrienol with PPAR**γ** antagonists synergistically inhibited MCF-7 and MDA-MB-231 cell growth. Combined treatment of **γ**-tocotrienol and PPAR**γ** agonists caused an increase in transcription activity of PPAR**γ** along with increased expression of PPAR**γ** and RXR, and decrease in PPAR**γ** coactivators, CBP p/300, CBP C-20, and SRC-1, in both breast cancer cell lines. In contrast, combined treatment of **γ**-tocotrienol with PPAR**γ** antagonists resulted in a decrease in transcription activity of PPAR**γ**, along with decreased expression of PPAR**γ** and RXR, increase in PPAR**γ** coactivators, and corresponding decrease in PI3K/Akt mitogenic signaling in these cells. These findings suggest that elevations in PPAR**γ** are correlated with increased breast cancer growth and survival, and treatment that decreases PPAR**γ** expression may provide benefit in the treatment of breast cancer.

## 1. Introduction

 Peroxisome proliferator activated receptor *γ* (PPAR*γ*) belongs to the nuclear receptor superfamily and functions as a ligand-activated transcription factor that forms a heterodimer complex with retinoid X receptor (RXR). This complex then binds to a specific DNA sequence called the peroxisome proliferator response element and initiates the recruitment of coactivator proteins such as CBP p/300, SRC-1, and CBP C-20, which further modulate gene transcription [1–3]. Studies have shown that PPAR*γ* is overexpressed in many types of breast cancer cells [4–7]. Experimental evidence in rodents has shown that overexpression of PPAR*γ* is associated with an increased incidence and growth in mammary tumors, whereas knockdown of PPAR*γ* expression was found to significantly inhibit spontaneous mammary tumor development [8, 9]. Taken together these results suggest that inhibition of PPAR*γ* expression and/or activity may be beneficial in the treatment of breast cancer. However, other studies have shown that treatment with the PPAR*γ* agonist rosiglitazone and troglitazone, or conversely with PPAR*γ* antagonists GW9662 and T0070907, were both found to significantly inhibit the growth of a wide variety of cancer cell lines [10, 11]. An explanation for these conflicting findings is not clearly evident, especially since some of the anticancer effects of these agents may be mediated through PPAR*γ*-independent mechanisms. Interpretation of these findings is further complicated by the fact that PPAR*γ* transcriptional activity can be modulated when phosphorylation by Akt and other kinases, which can occur from crosstalk with other mitogenic signaling pathways [12].

*γ*-Tocotrienol is a member of the vitamin E family of compounds that displays potent anticancer activity [13, 14]. The mechanism(s) involved in mediating the anticancer activity of *γ*-tocotrienol appear to involve the suppression of growth-factor-dependent mitogenic signaling, particularly the PI3K/Akt signaling pathway [15–18]. PI3K is a lipid signaling kinase that activates PDK-1, which subsequently phosphorylates and activates Akt. Activated Akt phosphorylates various proteins associated with cell proliferation and survival [19]. PDK-1 and Akt activity is terminated by phosphatases such as PTEN [20].

Recent studies have shown that tocotrienols activate specific PPARs in reporter-based assays [21], whereas other studies have shown that *γ*-tocotrienol increases intracellular levels of 15-lipoxygenase-2, the enzyme responsible for the conversion of arachidonic acid to the PPAR*γ* activating ligand, 15-S-hydroxyeicosatrienooic acid, in prostate cancer cells [22]. Therefore, it was hypothesized that the anticancer effects of *γ*-tocotrienol may be mediated, at least in part, through a PPAR*γ*-dependent mechanism. Studies were conducted to characterize the effects of *γ*-tocotrienol treatment alone and in combination with specific PPAR*γ* agonists and antagonists on the growth and survival of MCF-7 and MDA-MB-231 human breast cancer cells. Additional studies evaluated treatment effects on the expression of PPAR*γ* and PPAR*γ* coactivators, and PI3K/Akt mitogenic signaling in these breast cancer cell lines. Results from these studies further characterize the anticancer mechanism of action of *γ*-tocotrienol, as well as PPAR*γ* agonist and antagonists, and provides insights as to potential benefits of these therapies in the treatment of breast cancer. 

## 2. Materials and Methods

### 2.1. Reagents and Antibodies

All reagents were purchased from Sigma Chemical Company (St. Louis, MO) unless otherwise stated. Purified *γ*-tocotrienol (>98% purity) was generously provided as a gift by First Tech International Ltd (Hong Kong). The PPAR*γ* agonists, rosiglitazone and troglitazone, and the PPAR*γ* antagonists, GW9662 and T0070907, were purchased from Cayman Chemicals (San Diego, CA). Fetal bovine serum was purchased from American Type Culture Collection (Manassas, VA). Antibodies for *β*-actin, PPAR*γ*, Akt, phospho-Akt, PTEN, phospho-PTEN, PDK-1, PI3K, cleaved caspase-3, and cleaved PARP were purchased from Cell Signaling Technology (Beverly, MA). Antibodies for RXR, CBP C-20, SRC-1, and CBP p/300 were purchased from Santa Cruz Biotechnology (Santa Cruz, CA). Goat anti-rabbit and anti-mouse secondary antibodies were purchased from PerkinElmer Biosciences (Boston, MA).

### 2.2. Cell Lines and Culture Conditions

The estrogen-receptor negative MDA-MB-231, and the estrogen-receptor positive MCF-7 breast carcinoma cell lines were purchased from American Type Culture Collection (Manassas, VA). MDA-MB-231 and MCF-7 breast cancer cells were cultured in modified Dulbecco's modified Eagle Medium (DMEM)/F12 supplemented with 10% fetal bovine serum, 10 *μ*g/mL insulin, 100 U/mL penicillin, 0.1 mg/mL streptomycin at 37°C in an environment of 95% air and 5% CO_2_ in a humidified incubator. For subculturing, cells were rinsed twice with sterile Ca^2+^- and Mg^2+^-free phosphate-buffered saline (PBS) and incubated in 0.05% trypsin containing 0.025% EDTA in PBS for 5 min at 37°C. The released cells were centrifuged, resuspended in serum containing media, and counted using a hemocytometer.

### 2.3. Experimental Treatments

The highly lipophilic *γ*-tocotrienol was suspended in a solution of sterile 10% BSA as described previously [13, 14]. Briefly, an appropriate amount of *γ*-tocotrienol was first dissolved in 100 *μ*L of 100% ethanol, then added to a small volume of sterile 10% BSA in water and incubated overnight at 37°C with continuous shaking. This stock solution was then used to prepare various concentrations of treatment media. Stock solutions of rosiglitazone, troglitazone, GW9662 and T0070907 were prepared in DMSO. Ethanol and/or DMSO was added to all treatment media such that the final concentration was the same in all treatment groups within any given experiment and was always less than 0.1%.

### 2.4. Growth Studies

MCF-7 and MDA-MB-231 cells were plated at a density of 5 × 10^4^ cells/well (6 replicates/group) in 24 well culture plates and 1 × 10^4^ cells/well in 96 well culture plate, respectively and allowed to adhere overnight. The next day, cells were divided into different treatment groups, culture media was removed, washed with sterile PBS, then fed fresh media containing their respective treatments, and then returned to the incubator. Cells were treated with media containing 0–50 *μ*M rosiglitazone, troglitazone, GW9662, T0070907 or 0–8 *μ*M *γ*-tocotrienol alone or a combination for a 4-day culture period. Cells in each treatment group were fed fresh media every other day throughout the experimental period. For apoptosis experiments, MCF-7 and MDA-MB-231 cells were plated as described above. Cells were allowed to grow in control media for 3 days, after which they were exposed to the various treatments for a 24 h period. Treatment with 20 *μ*M *γ*-tocotrienol has previous been shown to induce apoptosis in breast cancer cells [13, 14] and was used as a positive control in this study.

### 2.5. Measurement of Viable Cell Number

MCF-7 and MDA-MB-231 viable cell number was determined using the 3-(4,5-dimethylthiazol-2yl)-2,5-diphenyl tetrazolium bromide (MTT) colorimetric assay as described previously [13, 14]. At the end of the treatment period, treatment media was removed and all cells were exposed for 3 h (96 well plates) or 4 h (24 well/plates) to fresh control media containing 0.41 mg/mL MTT at 37°C. Afterwards, media was removed and MTT crystals were dissolved in 1 mL of isopropanol for 24 culture plate or 100 *μ*L of DMSO for 96 culture plate assays. The optical density of each sample was measured at 570 nm at a microplate reader (Spectracount; Packard Bioscience Company, Meriden, CT) zeroed against a blank prepared from cell-free medium. The number of cells per well was calculated against a standard curve prepared by plating known cell densities, as determined by hemocytometer, in triplicate at the start of each experiment. 

### 2.6. Western Blot Analysis

MCF-7 and MDA-MB-231 cells were plated at a density of 1 × 10^6^ cells/100 mm culture dish and exposed to control or treatment media for a 4-day culture period. Afterwards, cells were washed with PBS, isolated with trypsin, and whole cell lysates were prepared in Laemmli buffer [23] as described previously [24]. The protein concentration in each sample was determined using Bio-Rad protein assay kit (Bio-Rad, Hercules, CA). Equal amounts of protein from each sample in a given experiment was loaded onto SDS-polyacrylamide minigels and electrophoresed through 5%–15% resolving gel. Proteins separated on each gel were transblotted at 30 V for 12–16 h at 4°C onto a polyvinylidene fluoride (PVDF) membrane (PerkinElmer Lifesciences, Wellesley, MA) in a Trans-Blot Cell (Bio-Rad, Hercules, CA) according to the method of Towbin et al. [25]. The membranes were then blocked with 2% BSA in 10 mM Tris HCl containing 50 mM NaCl and 0.1% Tween 20 pH 7.4 (TBST) and then incubated with specific primary antibodies against PPAR*γ*, Akt, phospho-Akt, PTEN, phospho-PTEN, PDK-1, PI3K, RXR, CBP C-20, SRC-1, CBP p/300, cleaved capase-3, cleaved PARP or *β*-actin, diluted 1 : 500 to 1 : 5000 in TBST/2% BSA for 2 h. Membranes are washed 5 times with TBST followed by incubation with the respective horseradish peroxide-conjugated secondary antibodies diluted 1 : 3000 to 1 : 5000 in TBST/2% BSA for 1 h followed by rinsing with TBST. Protein bands bound to the antibody were visualized by chemiluminescence (Pierce, Rockford, IL) according to the manufacturer's instructions and images were obtained using a Kodak Gel Logic 1500 Imaging System (Carestream Health Inc, Rochester, NY). The visualization of *β*-actin was performed to confirm equal sample loading in each lane. Images of protein bands on the film were acquired and scanning densitometric analysis was performed with Kodak molecular imaging software version 4.5 (Carestream Health Inc, Rochester, NY). All experiments were repeated at least three times and a representative western blot image from each experiment is shown in the figures.

### 2.7. Transient Transfection and Luciferase Reporter Assay

MCF-7 and MDA-MB-231 cells were plated at a density of 2 × 10^4^ per well in 96-well plates and allowed to adhere overnight. After this cells were transfected with 32 ng of PPRE X3-TK-luc (Addgene plasmid no. 1015) [26] and 3.2 ng of renilla luciferase plasmid per well (Promega, Madison, WI) using 0.8 *μ*L of lipofectamine 2000 transfection reagent for each well (Invitrogen, Grand Island, NY). After 6 h transfection, the media was removed; the cells were washed once and exposed to 100 *μ*L of control or treatment media for a 4-day culture period. Afterwards, cells were lysed with 75 *μ*L of passive lysis buffer and treated according to manufacturer's instructions using dual-glo luciferase assay system (Promega, Madison, WI). Luciferase activity of each sample was normalized by the level of renilla activity. Data is represented as mean fold changes in treated cells as compared to control cells. 

### 2.8. Statistical Analysis

The level of interaction between PPAR*γ* ligands and *γ*-tocotrienol was evaluated by isobologram method [27]. A straight line was formed by plotting IC_50_ doses of *γ*-tocotrienol and individual PPAR*γ* ligands on the *x*-axes and *y*-axes, respectively as determined by non-linear regression curve fit analysis using GraphPad Prism 4 (GraphPad Software inc. La Jolla, CA). The data point in the isobologram corresponds to the actual IC_50_ dose of combined *γ*-tocotrienol and PPAR*γ* ligands treatment. If a data point is on or near the line, this represents an additive treatment effect, whereas a data point that lies below or above the line indicates synergism or antagonism, respectively. Differences among the various treatment groups in growth studies and western blot studies were determined by analysis of variance followed by Dunnett's multiple range test. Differences were considered statistically significant at a value of *P* < 0.05.

## 3. Results

### 3.1. Antiproliferative Effects of *γ*-Tocotrienol, PPAR*γ* Agonists (Rosiglitazone and Troglitazone), and PPAR*γ* Antagonists (GW9662 and T0070907)

**Figure 1:**
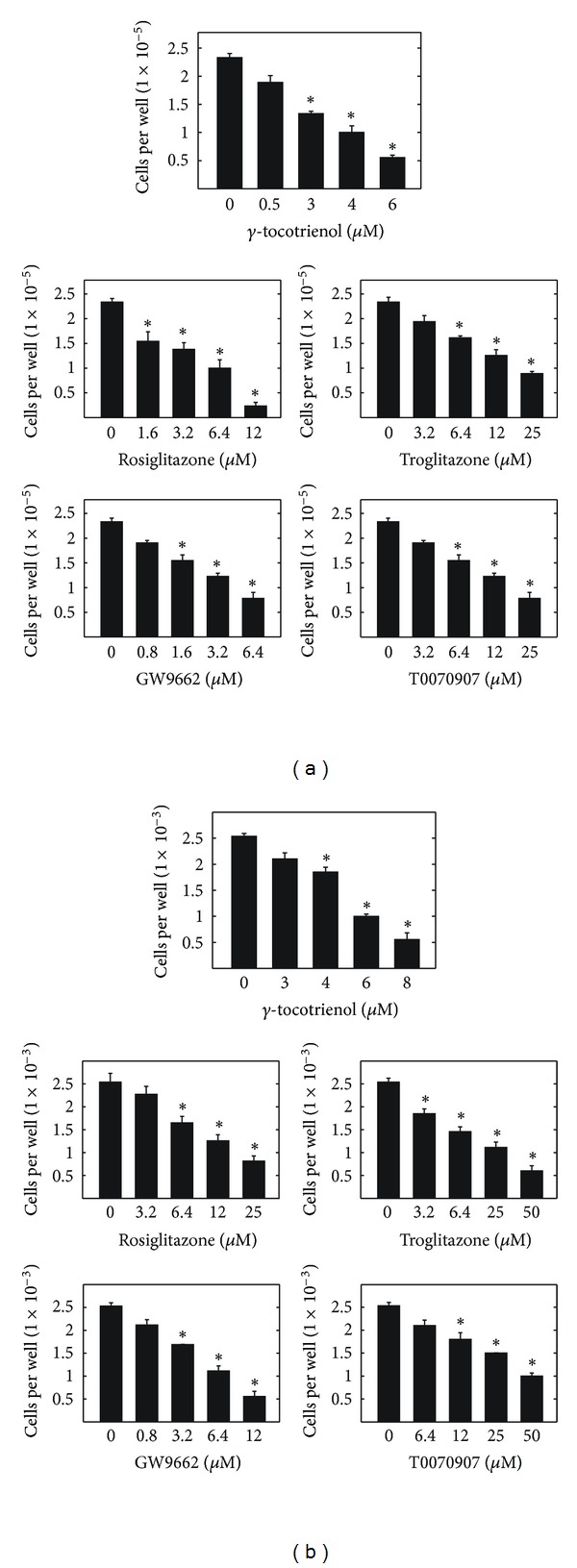
Antiproliferative effects of *γ*-tocotrienol, PPAR*γ* agonists (rosiglitazone and troglitazone), and PPAR*γ* antagonists (GW9662 and T0070907) on (a) MCF-7 and (b) MDA-MB-231 human breast cancer cells. MCF-7 cells were plated at a density of 5 × 10^4^ (6 wells per group) in 24-well culture plates and exposed to treatment media for a 4-day period. Afterwards viable cell number was determined using MTT colorimetric assay. MDA-MB-231 cells were plated at a density of 1 × 10^4^ (6 wells per group) in 96-well culture plates and exposed to treatment media for a 4-day period. Afterwards viable cell number was determined using MTT colorimetric assay. Vertical bars indicate mean cell count ± SEM in each treatment group. *P < 0.05 as compared with vehicle-treated controls.

Treatment with 3–6 *μ*M *γ*-tocotrienol, 1.6–12 *μ*M rosiglitazone, 6.4–25 *μ*M troglitazone, 1.6–6.4 *μ*M GW9662, or 6.4–25 *μ*M T0070907 was found to significantly inhibit growth of MCF-7 cells in a dose-responsive manner as compared to cells in the vehicle-treated control group (Figure 1(a)). Similarly, treatment with 4–8 *μ*M *γ*-tocotrienol, 6.4–25 *μ*M rosiglitazone, 3.2–50 *μ*M troglitazone, 3.2–12 *μ*M GW9662, and 12–50 *μ*M T0070907 significantly inhibited MDA-MB-231 cell growth in a dose-responsive manner as compared to cells in the vehicle-treated control group (Figure 1(b)).

### 3.2. Antagonistic Effects of PPAR*γ* Agonist Rosiglitazone and Troglitazone on the Antiproliferative Effects of *γ*-Tocotrienol

**Figure 2:**
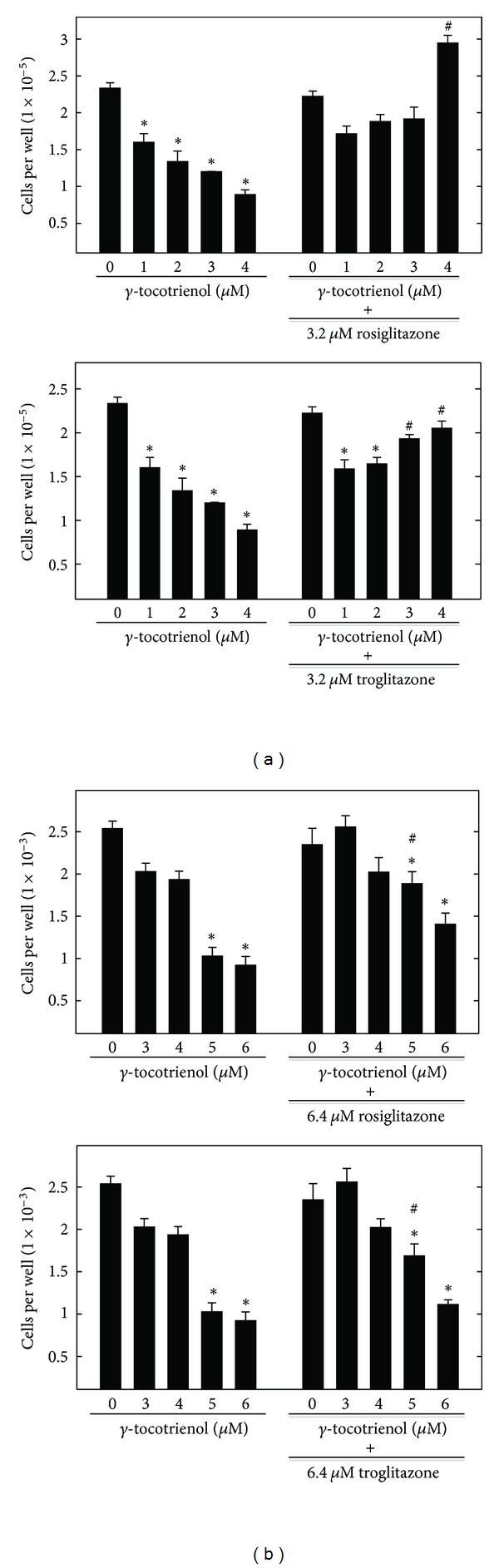
Effects of *γ*-tocotrienol, PPAR*γ* agonist rosiglitazone and troglitazone treatment alone or in combination on growth of (a) MCF-7 and (b) MDA-MB-231 human breast cancer cells. MCF-7 cells were initially plated at a density of 5 × 10^4^ (6 wells per group) in 24-well plates and (b) MDA-MB-231 were initially plated at a density of 1 × 10^4^ (6 wells per group) in 96-well culture plates and exposed to treatment media for a 4-day period. Afterwards, viable cell number was determined using MTT colorimetric assay. Vertical bars indicate the mean cell count ± SEM in each treatment group. *P < 0.05 as compared with vehicle-treated controls and ^#^ *P* < 0.05 as compared to their corresponding control treated with *γ*-tocotrienol alone.

Treatment with 1–6 *μ*M *γ*-tocotrienol alone significantly inhibited growth of MCF-7 (Figure 2(a)) and MDA-MB-231 (Figure 2(b)) breast cancer cells after a 4-day treatment period. However, the growth inhibitory effects of 1–4 *μ*M *γ*-tocotrienol on MCF-7 cells were reversed when given in combination with 3.2 *μ*M rosiglitazone or troglitazone (Figure 2(a), Top and Bottom). A similar, but less pronounced, reversal in 3–6 *μ*M *γ*-tocotrienol-induced growth inhibitory effects on MDA-MB-231 breast cancer cells was observed when used in combination with 6.4 *μ*M rosiglitazone or troglitazone (Figure 2(b), Top and Bottom). 

### 3.3. Enhancement of *γ*-Tocotrienol-Induced Antiproliferative Effects When Given in Combination with PPAR*γ* Antagonist GW9662 or T0070907

**Figure 3:**
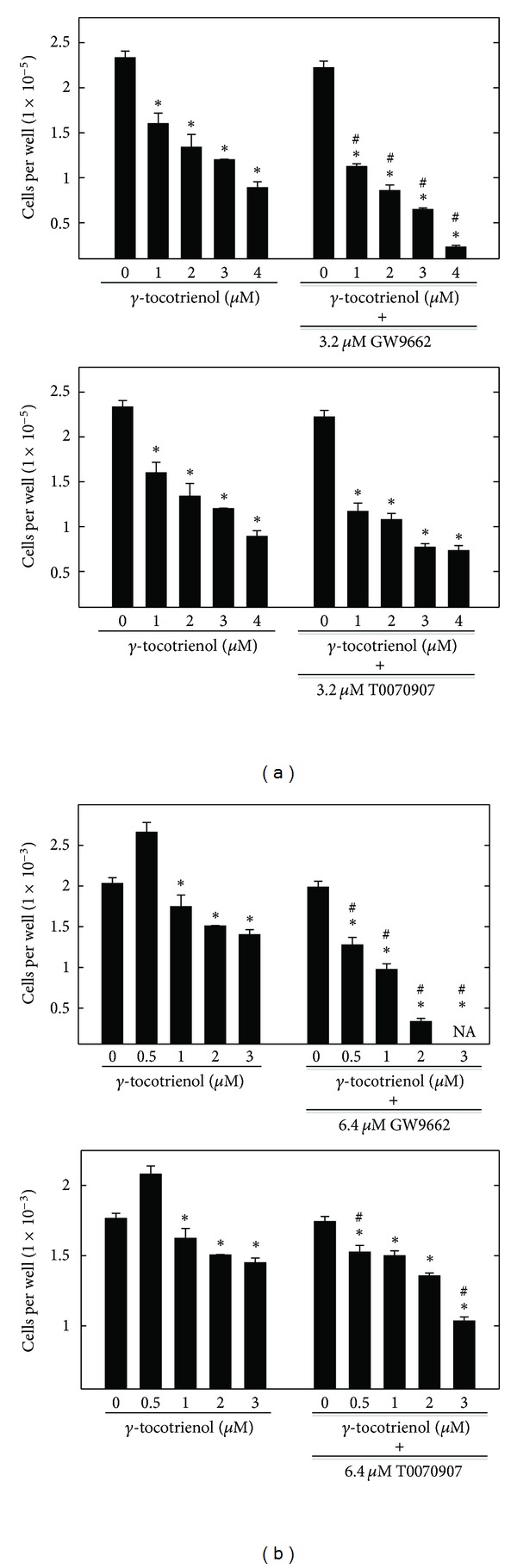
Effects of *γ*-tocotrienol, PPAR*γ* antagonists GW9662 and T0070907 treatment alone or in combination on growth of (a) MCF-7 and (b) MDA-MB-231 human breast cancer cells. MCF-7 cells were initially plated at a density of 5 × 10^4^ (6 wells per group) in 24-well plates and (b) MDA-MB-231 were initially plated at a density of 1 × 10^4^ (6 wells per group) in 96-well culture plates and exposed to treatment media for a 4-day period. Afterwards, viable cell number was determined using MTT colorimetric assay. Vertical bars indicate the mean cell count ± SEM in each treatment group. *P < 0.05 as compared with vehicle-treated controls and ^#^ *P* < 0.05 as compared to their corresponding control treated with *γ*-tocotrienol alone.

The growth inhibitory effects of 1–4 *μ*M *γ*-tocotrienol was significantly enhanced when given in combination with a subeffective dose (3.2 *μ*M) of the PPAR*γ* antagonist, GW9662, in MCF-7 breast cancer cells (Figure 3(a), Top). A slight, but insignificant enhancement of the growth inhibitory effects 1–4 *μ*M *γ*-tocotrienol was observed when combined with a subeffective dose (3.2 *μ*M) of the PPAR*γ* antagonist, T0070907, in MCF-7 breast cancer cells (Figure 3(a), Bottom). In MDA-MB-231 cells, 0.5–3 *μ*M*γ*-tocotrienol was used in combination with 6.4 *μ*M of the PPAR*γ* antagonists, GW9662 (Figure 3(b), Top) or T0070907 (Figure 3(b), Bottom) and was found to significantly enhanced the growth inhibitory effects of these agents. Higher dose ranges of *γ*-tocotrienol in combination with these same doses of PPAR*γ* antagonists resulted in a complete suppression in breast cancer cell growth such that viable cell number was undetectable using the MTT assay (data not shown).

### 3.4. Isobologram Analysis of Combined Treatment Effects of *γ*-Tocotrienol with PPAR*γ* Agonists and Antagonists

**Figure 4:**
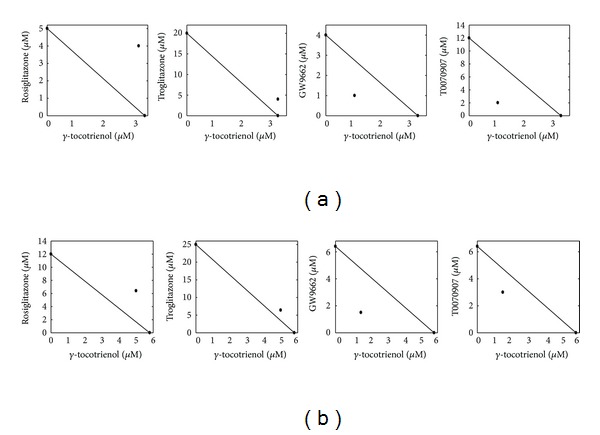
Isobologram analysis of combined treatment of *γ*-tocotrienol and PPAR*γ* ligands on (a) MCF-7 and (b) MDA-MB-231 human breast cancer cells. Individual IC_50_ doses for *γ*-tocotrienol, PPAR*γ* agonists (rosiglitazone and troglitazone), and PPAR*γ* antagonists (GW9662 and T0070907) were calculated and then plotted on the *x*-axes and *y*-axes, respectively. The data point on the isobologram represents the actual doses of combined *γ*-tocotrienol and PPAR*γ* ligands. Combined treatment of PPAR*γ* agonists rosiglitazone and troglitazone with *γ*-tocotrienol was found to be antagonistic, as evidenced by the location of the data point in the isobologram being well above the line defining additive effect. In contrast, the growth inhibitory effect of combined treatment of *γ*-tocotrienol with PPAR*γ* antagonists GW9662 and T0070907 was found to be synergistic, as evidenced by the location of the data point in the isobologram being well below the line defining additive effect for both cell lines.

Combined treatment of *γ*-tocotrienol with PPAR*γ* agonists, rosiglitazone, and troglitazone was found to be statistically antagonistic on MCF-7 (Figure 4(a)) and MDA-MB-231 (Figure 4(b)) breast cancer cell growth, as evidenced by the location of the data point in the isobologram being well above the line defining additive effect. In contrast, the growth inhibitory effect of combined treatment of *γ*-tocotrienol with PPAR*γ* antagonists, GW9662, and T0070907 were found to be statistically synergistic in both MCF-7 (Figure 4(a)) and MDA-MB-231 (Figure 4(b)) breast cancer cells, as evidenced by the location of the data point in the isobologram being well below the line defining additive effect. 

### 3.5. Effects of *γ*-Tocotrienol and PPAR*γ* Agonist Rosiglitazone and Troglitazone Given Alone or in Combination on PPAR*γ* and RXR Levels

**Figure 5:**
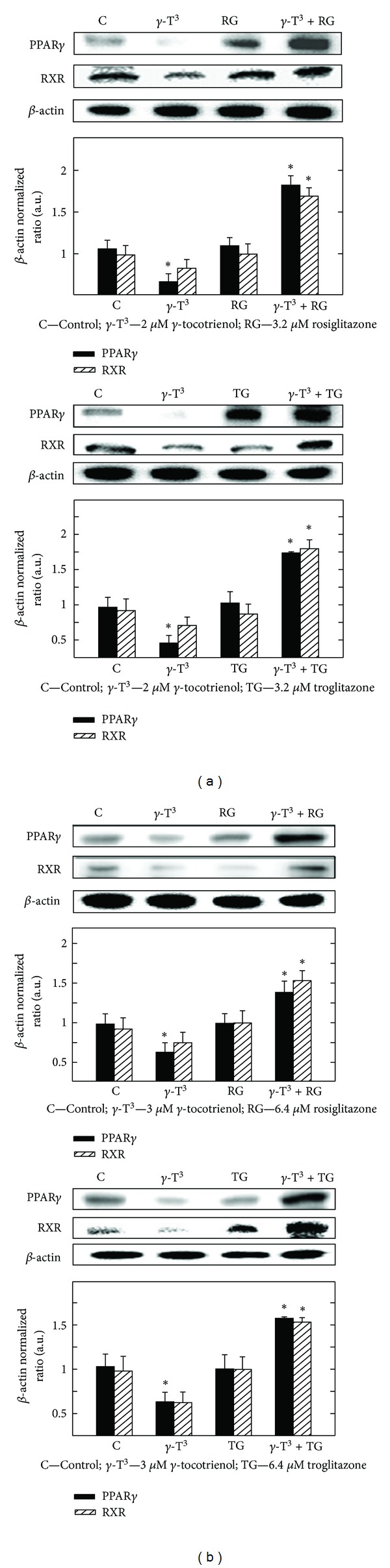
Western blot analysis of *γ*-tocotrienol and PPAR*γ* agonists (rosiglitazone and troglitazone) given alone or in combination on the levels of PPAR*γ* and RXR after a 4-day incubation period in (a) MCF-7 and (b) MDA-MB-231 human breast cancer cells. MCF-7 cells were initially plated at 1 × 10^6^ cells/100 mm culture dish and treated with control or treatment media containing 2 *μ*M *γ*-tocotrienol, 3.2 *μ*M rosiglitazone, or troglitazone alone or in combination. MDA-MB-231 cells were plated in a similar manner and treated with control or treatment media containing either 3 *μ*M *γ*-tocotrienol, 6.4 *μ*M rosiglitazone, or 6.4 *μ*M troglitazone alone or in combination. All cells were fed fresh treatment media every other day for 4-day incubation period. Afterwards, whole cell lysates were prepared for subsequent separation by polyacrylamide gel electrophoresis (50 *μ*g/lane) followed by Western blot analysis. Scanning densitometric analysis was performed on all blots done in triplicate and the integrated optical density of each band was normalized with corresponding *β*-actin, as shown in bar graphs below their respective Western blot images. Vertical bars in the graph indicate the normalized integrated optical density of bands visualized in each lane ± SEM. *P < 0.05 as compared with vehicle-treated controls.

Western blot analysis shows that treatment with 2 *μ*M (MCF-7 cells) or 3 *μ*M (MDA-MB-231 cells) *γ*-tocotrienol alone induced a decreased expression of PPAR*γ* and RXR as compared to the vehicle-treated controls (Figures 5(a) and 5(b)). Treatment with 3.2 *μ*M rosiglitazone or troglitazone alone in MCF-7 cells or 6.4 *μ*M rosiglitazone or troglitazone alone in MDA-MB-231 cells had little or no effect on PPAR*γ* or RXR levels (Figures 5(a) and 5(b)). However, combined treatment with similar doses of *γ*-tocotrienol and rosiglitazone or troglitazone resulted in a significant increase in PPAR*γ* and RXR expression in both MCF-7 and MDA-MB-231 breast cancer cell lines (Figures 5(a) and 5(b)). 

### 3.6. Effects of *γ*-Tocotrienol and PPAR*γ* Antagonist GW9662 and T0070907 Given Alone or in Combination on PPAR*γ* and RXR Levels

**Figure 6:**
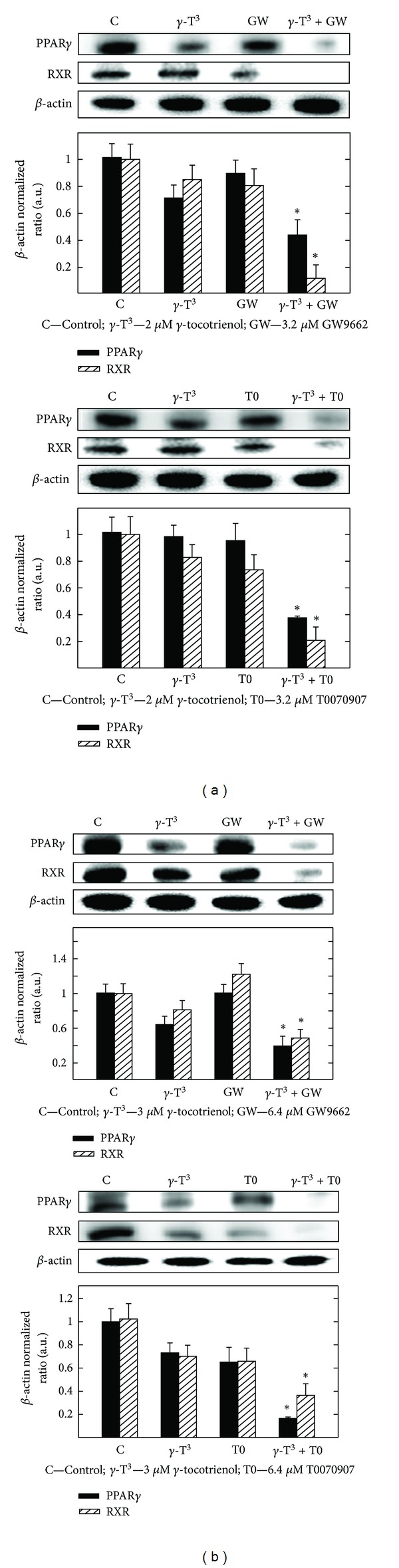
Western blot analysis of *γ*-tocotrienol and PPAR*γ* antagonists (GW9662 and T0070907) given alone or in combination on the levels of PPAR*γ* and RXR after a 4-day incubation period in (a) MCF-7 and (b) MDA-MB-231 cells. MCF-7 cells were initially plated at 1 × 10^6^ cells/100 mm culture dish and treated with control or treatment media containing either 2 *μ*M *γ*-tocotrienol, 3.2 *μ*M GW9662, or T0070907 alone or in combination. MDA-MB-231 cells were plated in a similar manner and treated with control or treatment media containing either 3 *μ*M *γ*-tocotrienol, 6.4 *μ*M GW9662, or 6.4 *μ*M T0070907 alone or in combination. All cells were fed fresh treatment media every other day for 4-day incubation period. Afterwards, whole cell lysates were prepared from each treatment group for subsequent separation by polyacrylamide gel electrophoresis (50 *μ*g/lane) followed by Western blot analysis. Scanning densitometric analysis was performed on all blots done in triplicate and the integrated optical density of each band was normalized with corresponding *β*-actin, as shown in bar graphs below their respective Western blot images. Vertical bars in the graph indicate the normalized integrated optical density of bands visualized in each lane ± SEM. *P < 0.05 as compared with vehicle-treated controls.

Western blot analysis shows that treatment with 2 *μ*M (MCF-7 cells) or 3 *μ*M (MDA-MB-231 cells) *γ*-tocotrienol alone induced decrease expression of PPAR*γ* and RXR as compared to the vehicle-treated controls (Figures 6(a) and 6(b)). Treatment with 3.2 *μ*M (MCF-7 cells) or 6.4 *μ*M (MDA-MB-231 cells) of the PPAR*γ* antagonists, GW9662 or T0070907 alone had only slight effects on PPAR*γ* and RXR expression (Figures 6(a) and 6(b)). However, combined treatment with these same doses of *γ*-tocotrienol and GW9662 or T0070907 caused a significant reduction in PPAR*γ* and its heterodimer partner, RXR, in both MCF-7 and MDA-MB-231 cells as compared to vehicle treated controls (Figures 6(a) and 6(b)).

### 3.7. Effects of *γ*-Tocotrienol and PPAR*γ* Agonist Rosiglitazone and Troglitazone Given Alone or in Combination on PPRE Mediated Reporter Activity

**Figure 7:**
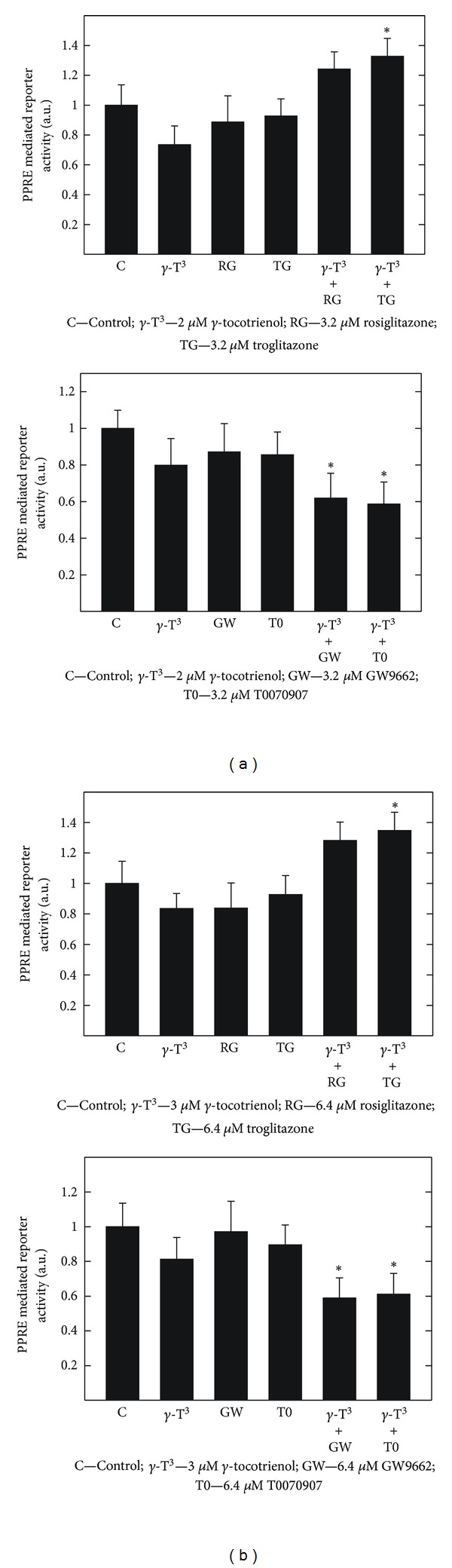
Luciferase assay was performed on (a) MCF-7 and (b) MDA-MB-231 human breast cancer cells. The cells were initially plated at a density of 2 × 10^4^ cells/well in 96-well plates. Cells were then transfected by adding 32 ng of PPRE X3-TK-luc and 3.2 ng of renilla luciferase plasmid in 0.8 *μ*L of lipofectamine 2000 transfection reagent. Following a 6-h incubation period, MCF-7 cells were treated with control or treatment media containing 0–2 *μ*M *γ*-tocotrienol, 0–3.2 *μ*M rosiglitazone, 0–3.2 *μ*M troglitazone, 0–3.2 *μ*M GW9662, or 0–6.4 *μ*M T0070907 alone or in combination. MDA-MB-231 cells were initially plated in a similar manner and treated with control or treatment media containing 0–3 *μ*M *γ*-tocotrienol, 0–6.4 *μ*M rosiglitazone, 0–6.4 *μ*M troglitazone, 0–6.4 *μ*M GW9662, or 0–6.4 *μ*M T0070907 alone or in combination. All cells were fed fresh treatment media every other day for 4-day incubation period. Afterwards, cells were lysed with 75 *μ*L of passive lysis buffer and treated according to manufacturer's instructions using the dual-glo luciferase assay system. Results were calculated as raw luciferase units divided by raw renilla units. Vertical bars indicate PPRE mediated reporter activity ± SEM (arbitrary units) in each treatment group. *P < 0.05 as compared with vehicle-treated controls.

Luciferase assay shows that the treatment with 2 *μ*M (MCF-7 cells) or 3 *μ*M (MDA-MB-231 cells) *γ*-tocotrienol alone induced only slight effects in the PPRE mediated reporter activity as compared to vehicle treated controls (Figures 7(a) and 7(b), Top and Bottom). Treatment with 3.2 *μ*M (MCF-7 cells) or 6.4 *μ*M (MDA-MB-231 cells) with the PPAR*γ* agonists, rosiglitazone, and troglitazone, or PPAR*γ* antagonists, GW9662 and T0070907, alone, caused a slight, but insignificant decrease in PPRE mediated reporter activity (Figures 7(a) and 7(b), Top and Bottom). However, combined treatment with these same doses of *γ*-tocotrienol and rosiglitazone or troglitazone caused an increase in transcription activity of PPAR*γ* in both MCF-7 and MDA-MB-231 cells as compared to vehicle-treated controls (Figures 7(a) and 7(b), Top). In contrast, combined treatment with these same doses of *γ*-tocotrienol and GW9662 or T0070907 caused a significant decrease PPRE mediated reporter activity in both MCF-7 and MDA-MB-231 cells as compared to vehicle-treated controls (Figures 7(a) and 7(b), Bottom).

### 3.8. Effects of *γ*-Tocotrienol and PPAR*γ* Agonist Rosiglitazone and Troglitazone Given Alone or in Combination on Coactivator Expression

**Figure 8:**
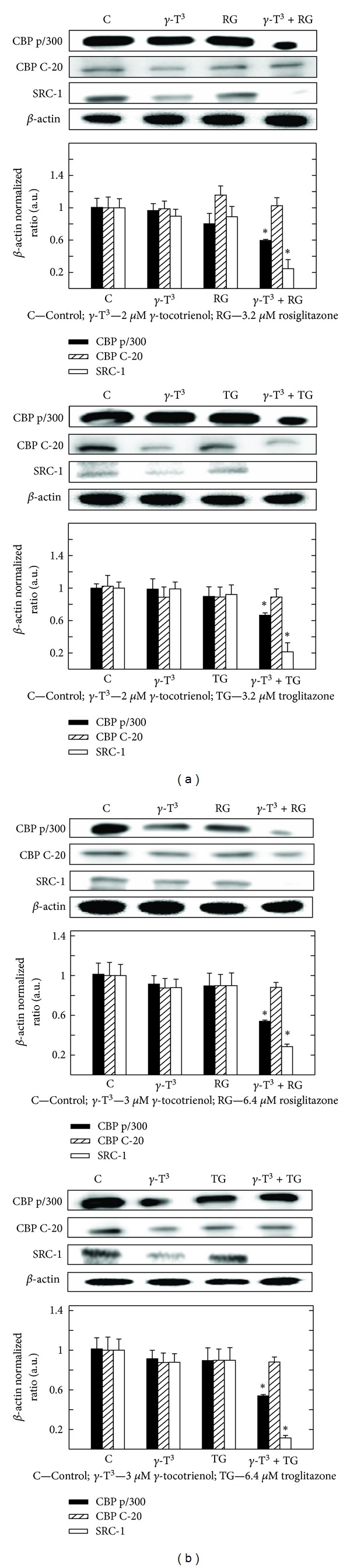
Western blot analysis of *γ*-tocotrienol and PPAR*γ* agonists (rosiglitazone and troglitazone) when used alone or in combination on the levels of CBP p/300, CBP C-20, and SRC-1 in (a) MCF-7 and (b) MDA-MB-231 human breast cancer cells. MCF-7 cells were initially plated at 1 × 10^6^ cells/100 mm culture dish and treated with control or treatment media containing 2 *μ*M *γ*-tocotrienol, 3.2 *μ*M rosiglitazone, or 3.2 *μ*M troglitazone alone or in combination. MDA-MB-231 cells were plated in a similar manner and treated with control or treatment media containing either 3 *μ*M *γ*-tocotrienol or 6.4 *μ*M rosiglitazone or 6.4 *μ*M troglitazone alone or in combination. All cells were fed fresh treatment media every other day for a 4-day incubation period. Afterwards, whole cell lysates were from cell in each treatment group and prepared for subsequent separation by polyacrylamide gel electrophoresis (50 *μ*g/lane) followed by Western blot analysis. Scanning densitometric analysis was performed on all blots done in triplicate and the integrated optical density of each band was normalized with corresponding *β*-actin, as shown in bar graphs below their respective Western blot images. Vertical bars in the graph indicate the normalized integrated optical density of bands visualized in each lane ± SEM. *P < 0.05 as compared with vehicle-treated controls.

Western blot analysis shows that treatment with 2 *μ*M (MCF-7 cells) or 3 *μ*M (MDA-MB-231 cells) *γ*-tocotrienol alone induced only slight, but insignificant effects in the expression of CBP p/300, CBP C-20, or SRC-1 as compared to the vehicle-treated controls (Figures 8(a) and 8(b)). Treatment with 3.2 *μ*M (MCF-7 cells) or 6.4 *μ*M (MDA-MB-231 cells) with the PPAR*γ* agonists, rosiglitazone and troglitazone alone caused a slight decrease in CBP p/300 and SRC-1, but not CBP C-20, expression (Figures 8(a) and 8(b)). However, combined treatment with these same doses of *γ*-tocotrienol and rosiglitazone and troglitazone cause a significant decrease in CBP p/300, CBP C-20, or SRC-1 expression in both MCF-7 and MDA-MB-231 cells as compared to vehicle treated controls (Figures 8(a) and 8(b)). 

### 3.9. Effects of *γ*-Tocotrienol and PPAR*γ* Antagonist GW9662 and T0070907 Given Alone or in Combination on Coactivator Expression

**Figure 9:**
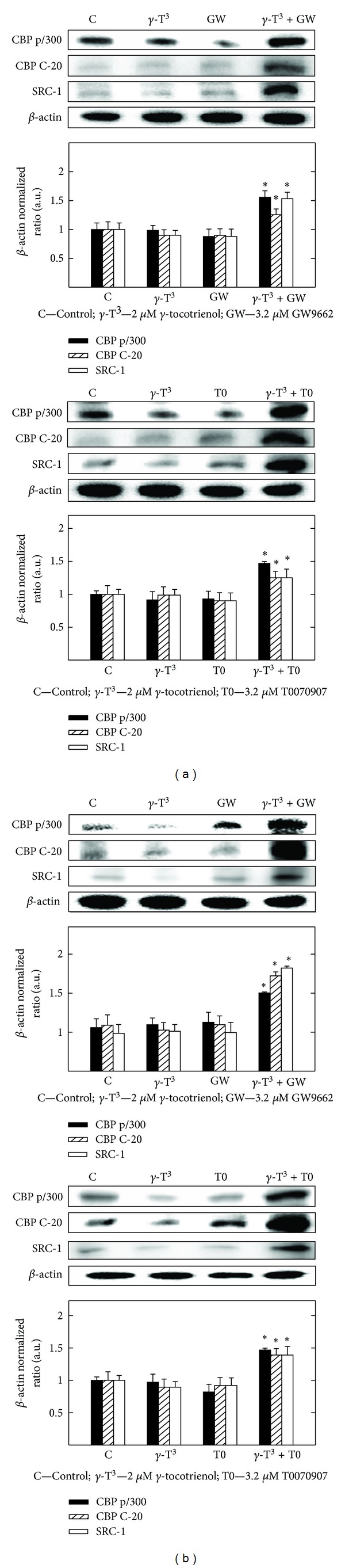
Western blot analysis of *γ*-tocotrienol and PPAR*γ* antagonists (GW9662 and T0070907) when used alone or in combination with each other to determine protein levels of CBP p/H300, CBP C-20, and SRC-1 in (a) MCF-7 and (b) MDA-MB-231 cells. MCF-7 cells were initially plated at 1 × 10^6^ cells/100 mm culture plate and treated with control or treatment media containing either 2 *μ*M *γ*-tocotrienol, 3.2 *μ*M GW9662, or 3.2 *μ*M T0070907 alone and in combination. MDA-MB-231 cells were plated in a similar manner and cells were treated with control or treatment media containing 3 *μ*M *γ*-tocotrienol, 6.4 *μ*M GW9662, or 6.4 *μ*M T0070907 alone or in combination. All cells were fed fresh treatment media every other day for a 4-day incubation period. Afterwards, whole cell lysates were prepared from cells in each treatment group for subsequent separation by polyacrylamide gel electrophoresis (50 *μ*g/lane) followed by Western blot analysis. Scanning densitometric analysis was performed on all blots done in triplicate and the integrated optical density of each band was normalized with corresponding *β*-actin, as shown in bar graphs below their respective Western blot images. Vertical bars in the graph indicate the normalized integrated optical density of bands visualized in each lane ± SEM. *P < 0.05 as compared with vehicle-treated controls.

Western blot analysis shows that treatment with 2 *μ*M (MCF-7 cells) or 3 *μ*M (MDA-MB-231 cells) *γ*-tocotrienol alone induced only slight effects in the expression of CBP p/300, CBP C-20, or SRC-1 as compared to the vehicle-treated controls (Figures 9(a) and 9(b)). Treatment with 3.2 *μ*M (MCF-7 cells) or 6.4 *μ*M (MDA-MB-231 cells) of the PPAR*γ* antagonists, GW9662 and T0070907, alone had only slight effects on CBP p/300, CBP C-20, or SRC-1 expression (Figures 9(a) and 9(b)). However, combined treatment with these same doses of *γ*-tocotrienol and rosiglitazone and troglitazone cause a significant increase in CBP p/300, CBP C-20, or SRC-1 expression in both MCF-7 and MDA-MB-231 cells as compared to vehicle-treated controls (Figures 9(a) and 9(b)). 

### 3.10. Effects of *γ*-Tocotrienol and PPAR*γ* Antagonist GW9662 and T0070907 Given Alone or in Combination on PI3K/Akt Mitogenic Signaling

**Figure 10:**
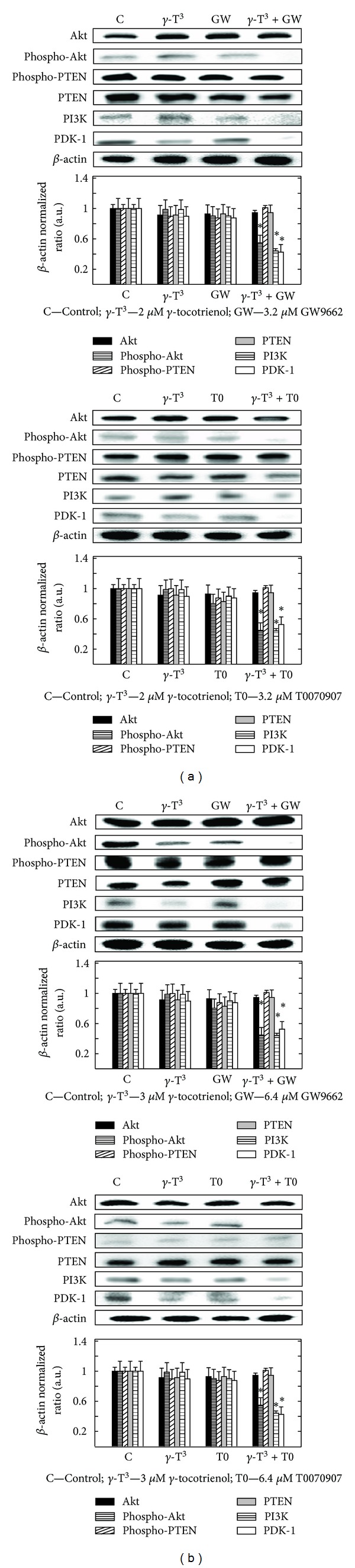
Western blot analysis of *γ*-tocotrienol and PPAR*γ* antagonists (GW9662 or T0070907) alone or in combination on Akt, phospho-Akt, PTEN, phospho-PTEN, PI3K, and PDK-1 levels on (a) MCF-7 and (b) MDA-MB-231 cells. MCF-7 cells were initially plated at 1 × 10^6^ cells/100 mm culture dish and treated with control or treatment media containing 2 *μ*M *γ*-tocotrienol, 3.2 *μ*M GW9662, or 3.2 *μ*M T0070907 alone or in combination. MDA-MB-231 cells were also plated in a similar manner and cells were treated with control or treatment media containing 3 *μ*M *γ*-tocotrienol, 6.4 *μ*M GW9662, or 6.4 *μ*M T0070907 alone or in combination. All cells were fed fresh treatment media every other day for a 4-day incubation period. Afterwards, whole cell lysates were prepared from cells in each treatment group for subsequent separation by polyacrylamide gel electrophoresis (50 *μ*g/lane) followed by Western blot analysis. Scanning densitometric analysis was performed on all blots done in triplicate and the integrated optical density of each band was normalized with corresponding *β*-actin, as shown in bar graphs below their respective Western blot images. Vertical bars in the graph indicate the normalized integrated optical density of bands visualized in each lane ± SEM. *P < 0.05 as compared with vehicle-treated controls.

Treatment of 2 *μ*M *γ*-tocotrienol with 3.2 *μ*M of the PPAR*γ* antagonists GW9662 or T0070907 alone had little or no effects on intracellular levels of Akt, phospho-Akt, PTEN, phospho-PTEN, PI3K, and PDK-1 in MCF-7 cells after a 4-day treatment period (Figure 10(a)). However, combined treatment with the same doses of these agents caused a significant decrease in levels of phospho-Akt, PDK-1, and PI3K, but had little or no effect on total Akt and PTEN, and phospho-PTEN levels as compared to MCF-7 cells in the vehicle-treated control groups (Figure 10(a)). Similarly, treatment of 3 *μ*M *γ*-tocotrienol, 6.4 *μ*M GW9662 or 6.4 *μ*M T0070907 alone had little or no effect on intracellular levels of phospho-Akt (activated), PDK-1, PI3K, Akt, PTEN, and phospho-PTEN in MDA-MB-231 breast cancer cells, as compared to vehicle-treated controls (Figure 10(b)). Combined treatment with the same doses of these agents resulted in a significant decrease in phospho-Akt, PDK-1, and PI3K levels as compared to MDA-MB-231 breast cancer cells in the vehicle-treated control group (Figure 10(b)). 

Similar studies were conducted to determine the effects of combined *γ*-tocotrienol treatment with PPAR*γ* agonist rosiglitazone and troglitazone on PI3K/Akt mitogenic signaling in MCF-7 and MDA-MB-231 breast cancer cells. However, little or no differences in the relative levels of these mitogenic proteins were observed among the different treatment groups (data not shown), apparently because cells in the various treatment groups were actively proliferating at a near maximal growth rate.

### 3.11. Apoptotic Effects of *γ*-Tocotrienol and PPAR*γ* Antagonist GW9662 and T0070907 Given Alone or in Combination

**Figure 11:**
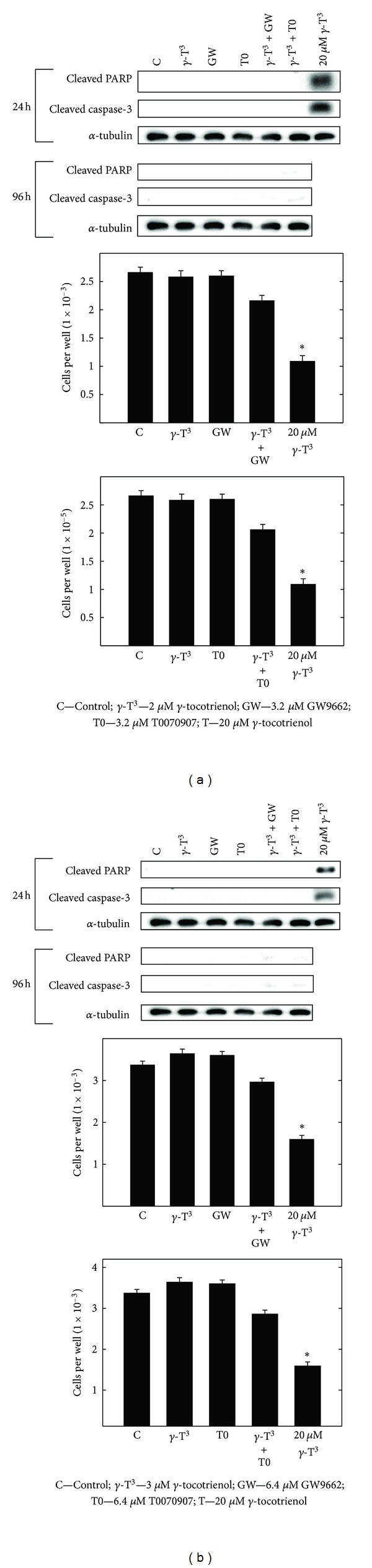
Apoptotic effects of *γ*-tocotrienol and PPAR*γ* antagonists (GW9662 or T0070907) alone or in combination on caspase-3 and cleaved PARP levels on (a) MCF-7 and (b) MDA-MB-231 human breast cancer cells. For Western blot studies, MCF-7 and MDA-MB-231 cells were initially plated at 1 × 10^6^ cells/100 mm culture dish and maintained on control media for a 3-day culture period. Afterwards, cells were divided into the various treatment groups, media was removed, and cells were exposed to their respective treatment media for a 24-h treatment period. In addition, cells were exposed to their respective treatment media for a 96-h treatment period, where fresh media was added every other day. MCF-7 cells were exposed to treatment media containing 0–2 *μ*M *γ*-tocotrienol, 0–3.2 *μ*M GW9662, or 0–3.2 *μ*M T0070907 alone or in combination, whereas MDA-MB-231 cells exposed to treatment media containing 0–3 *μ*M *γ*-tocotrienol, 0–6.4 *μ*M GW9662, or 0–6.4 *μ*M T0070907 alone or in combination. Afterwards, whole cell lysates were prepared from cells in each treatment group for subsequent separation by polyacrylamide gel electrophoresis (50 *μ*g/lane) followed by western blot analysis. In parallel studies, (a) MCF-7 cells were plated at a density of 5 × 10^4^ (6 wells per group) in 24-well culture plates, whereas (b) MDA-MB-231 cells were plated at a density of 1 × 10^4^ (6 wells per group) in 96-well culture plates and exposed to the same treatments as described above. After a 24-h treatment exposure, viable cell number in all treatment groups was determined using MTT assay. Vertical bars indicate the mean cell count ± SEM in each treatment group. *P < 0.05 as compared with vehicle-treated controls.

In order to determine if the growth inhibitory effects resulting from combined treatment with subeffective doses of *γ*-tocotrienol and PPAR*γ* antagonists might result from a reduction in viable cell number, studies were conducted to determine the acute effects (24-h) and chronic effects (96-h) of these treatment on the initiation of apoptosis and cell viability. Western blot analysis shows that treatment with 2 *μ*M (MCF-7 cells) or 3 *μ*M (MDA-MB-231 cells) *γ*-tocotrienol alone had no effect on the expression of cleaved PARP, cleaved caspase-3 or viable cell number after a 24-h and 96-h treatment exposure (Figures 11(a) and 11(b)). Treatment with 3.2 *μ*M (MCF-7 cells) or 6.4 *μ*M (MDA-MB-231 cells) of the PPAR*γ* antagonists, GW9662 and T0070907, alone, or in combination with their respective treatment dose of *γ*-tocotrienol was also found to have no effect on the expression of cleaved PARP, cleaved caspase-3 or viable cell number 24-h after treatment exposure (Figures 11(a) and 11(b)). However, treatment with 20 *μ*M *γ*-tocotrienol, a dose previously shown to induce apoptosis in mammary cancer cells [13, 14] and used as an apoptosis-inducing positive control in this experiments was found to induce a large increase in cleaved PARP and cleaved caspase-3 levels, and corresponding decrease in viable cell number in both MCF-7 and MDA-MB-231 breast cancer cells 24 h following treatment exposure (Figures 11(a) and 11(b)). The positive apoptosis control treatment of 20 *μ*M *γ*-tocotrienol was not included in the 96 h treatment exposure experiment, because by the end of this experiment there are no viable cells remaining in this treatment group.

## 4. Discussion

Results in these studies demonstrate that when given alone, treatment with *γ*-tocotrienol, PPAR*γ* agonists (rosiglitazone and troglitazone), or PPAR*γ* antagonists (GW9662 and T0070907), all induce a significant dose-responsive inhibition in the growth of MCF-7 and MDA-MB-231 human breast cancer cells in culture. However, when used in combination, treatment with low doses of PPAR*γ* agonists were found to reverse, whereas treatment with low doses of PPAR*γ* antagonists were found to synergistically enhance the antiproliferative effects of *γ*-tocotrienol. Additional studies determined that the synergistic inhibition of MCF-7 and MDA-MB-231 tumor cell growth resulting from combined low dose treatment of *γ*-tocotrienol with PPAR*γ* antagonists was associated with a reduction in PPAR*γ*, PPRE mediated reporter activity, and RXR, an increase in PPAR*γ* coactivator expression, and a corresponding suppression in PI3K/Akt mitogenic-signaling. Conversely, enhancement in MCF-7 and MDA-MB-231 tumor cell growth resulting from combined low dose treatment of *γ*-tocotrienol with PPAR*γ* agonists was associated with an increase in PPAR*γ*, PPRE mediated reporter activity, and RXR, a decrease in PPAR*γ* coactivator expression, and a corresponding restoration in EGF-dependent PI3K/Akt mitogenic-signaling as compared to their vehicle-treated control group. Taken together, these finding demonstrate that combined treatment of *γ*-tocotrienol with PPAR*γ* antagonists display synergistic anticancer activity and may provide some benefit in the treatment of human breast cancer. These finding also demonstrate the importance of matching complimentary anticancer agents for use in combination therapy because a mismatch may result in an antagonistic and undesirable therapeutic response. 

Previous investigations have shown that both PPAR*γ* agonists and antagonists act as effective anticancer agents [28, 29]. The role of PPAR*γ* agonists as anticancer agents has been well characterized in treatment of colon, gastric, and lung cancer [3, 11], whereas, PPAR*γ* antagonists have been shown to induce potent antiproliferative effects in many hematopoietic and epithelial cancer cell lines [11, 28]. Results in the present study confirm and extend these previous findings. Dose-response studies showed that treatment with either PPAR*γ* agonist or antagonist significantly inhibited the growth of human MCF-7 and MDA-MB-231 breast cancer cells in culture. Furthermore, treatment-induced antiproliferative effects were found to be more pronounce in MDA-MB-231 as compared to MCF-7 breast cancer cells, and these results are similar to those previously reported [28]. 

Numerous investigations have established that *γ*-tocotrienol acts as a potent anticancer agent that inhibits the growth of mouse [16, 30] and human [31, 32] breast cancer cells. Furthermore, studies have also shown that combined treatment of *γ*-tocotrienol with other traditional chemotherapies often results in an additive or synergistic inhibition in cancer cell growth and viability [16, 30]. The rationale for using tocotrienols in combination therapy is based on the principle that resistance to a single agent can be overcome with the use of multiple agents that display complimentary anticancer mechanisms of action. Initial studies showed the additive anticancer effects of mixed tocotrienols and tamoxifen on growth of the estrogen receptor positive MCF-7 and the estrogen receptor negative MDA-MB-435 cells [33] and these findings were later confirmed in other reports [34]. Recent studies have also shown synergistic anticancer effects of combined use *γ*-tocotrienol with statins [35–37], tyrosine kinase inhibitors [18, 38], COX-2 inhibitors [39, 40], and cMet inhibitors [41]. These studies concluded that combination therapy is most effective when the anticancer mechanism of action of *γ*-tocotrienol compliments the mechanism of action of the other drug, and may provide significant health benefits in the prevention and/or treatment of breast cancer in women, while at the same time avoiding tumor resistance or toxic effects that is commonly associated with high-dose monotherapy.

The exact role of PPAR*γ* in breast cancer cell proliferation and survival is not clearly understood. Previous studies have suggested that PPAR*γ* activation results in extensive accumulation of lipids and changes in mammary epithelial cell gene expression that promotes a more differentiated and less malignant phenotype, and attenuates breast cancer cell growth and progression [42, 43]. Other studies have shown that *γ*-tocotrienol enhances the expression of multiple forms of PPARs by selectively regulating PPAR target genes [21]. The antiproliferative effects of *γ*-tocotrienol have been previously hypothesized to be mediated by the action of *γ*-tocotrienol to stimulate PPAR*γ* activation by increasing the production of the PPAR*γ* ligand, 15-lipoxygenase-2, in human prostate cancer cells [22]. However, findings in the present study using two distinct types of human breast cancer cell lines showed that low-dose treatment with *γ*-tocotrienol decreased PPAR*γ* levels, whereas combined treatment of *γ*-tocotrienol with PPAR*γ* agonists resulted in an elevation in PPAR*γ* levels and a corresponding increase in breast cancer cell growth. These contradictory findings might be explained by differences in the cancer cell types and experimental models used to examine combination treatment effects in these different studies. Nevertheless, the present finding clearly demonstrate an antagonistic effect on breast cancer cell proliferation when treated with the combination of *γ*-tocotrienol and PPAR*γ* agonists, and provides strong evidence that increased expression of PPAR*γ* is a negative indicator for breast cancer responsiveness to anticancer therapy. This hypothesis is further evidence by the finding that PPAR*γ* expression is elevated in breast cancer cells as compared to normal mammary epithelial cells [9, 44], and mice genetically predisposed to developing mammary tumors constitutively express high levels of activated PPAR*γ* as compared to control mice [9, 44]. It is also possible that the anticancer effects of high-dose treatment with PPAR*γ* agonists may be mediated through PPAR*γ*-independent mechanisms.

The present study also confirms and extends previous findings showing that treatment with PPAR*γ* antagonists significantly inhibits growth of breast cancer cells. Experimental results showed that PPAR*γ* antagonist downregulate PPAR*γ* activation and expression and these effects were associated with enhanced responsiveness to anticancer therapy [45, 46]. However, the present study also shows that combined treatment of *γ*-tocotrienol with PPAR*γ* antagonist induced a relative large decrease in transcription activity of PPAR*γ*. This treatment was also shown to result in decreased expression of PPAR*γ* and RXR, and these effects were associated with a significant decrease in breast cancer cell growth. PPAR*γ* functions as a heterodimer with its obligate heterodimer partner-RXR. Like other nuclear hormone receptors, the PPAR*γ*-RXR heterodimer recruits cofactor complexes, either coactivators or corepressors to modulate their transcriptional activity [45]. Upon binding of a ligand to the heterodimer complex, corepressors are displaced and the receptor then associates with a coactivator molecule. These coactivators include SRC-1, CBP C-20, and the CBP homologue p/300 [47, 48]. Combined treatment of *γ*-tocotrienol and PPAR*γ* antagonists-induced suppression of transcription of PPAR*γ*, appears to also decrease the recruitment of coactivator molecules to available PPAR*γ*-RXR heterodimers for translocation into the nucleus, and ultimately resulting in an elevation of free coactivator levels in the cytoplasm. Taken together these results suggest that breast cancer cells require PPAR*γ* activation for their survival, and that treatments designed to reduce or inhibition of PPAR*γ* levels and/or activation and may provide an effective strategy in treatment of breast cancer.

PPAR*γ* activity can be modulated by phosphorylation at multiple sites [49]. In addition, PPAR*γ* ligands can reduce the activity of PI3K and its downstream target Akt [50]. Combined treatment of *γ*-tocotrienol with PPAR*γ* antagonists was found to reduced PI3K, phosphorylated PDK-1 (active), and phosphorylated-Akt (active) levels in MCF-7 and MDA-MB-231 breast cancer cells. Furthermore, these effects were not associated with an increase in PTEN activity, the phosphatase involved in the inactivation of PDK and Akt. These findings indicate that the antiproliferative effects of combined *γ*-tocotrienol and PPAR*γ* antagonists treatment is mediated through a suppression in PI3K/Akt mitogenic signaling. These effects were found to be cytostatic in nature, and not associated with a decrease in cell viability resulting from the initiation of apoptosis. Previous findings have also shown that treatment with PPAR*γ* antagonists can cause a decrease in PI3K/Akt mitogenic signaling [51]. 

## 5. Conclusion

Result in these studies demonstrate that combined low-dose treatment of *γ*-tocotrienol and PPAR*γ* antagonists act synergistically to inhibit human breast cancer cell proliferation, and this effect appears to be mediated by a large reduction in PPAR*γ* expression and corresponding reduction in PI3K/Akt mitogenic signaling. Although high dose treatment with PPAR*γ* agonist also was also found to inhibit human breast cancer cells growth, it is most likely that these effects are mediated through PPAR*γ*-independent mechanisms because the preponderance of experimental evidence strongly suggest that elevations in PPAR*γ* expression is an indicator of robust breast cancer cell growth and resistance to anticancer therapy, whereas a reduction in PPAR*γ* expression is an indicator of decreased breast cancer proliferation and increased responsiveness to chemotherapeutic agents. These findings also show that combination anticancer therapy does not always result in an additive or synergistic anticancer response, but can result in a paradoxical/antagonistic response as was observed with the combined treatment of *γ*-tocotrienol with PPAR*γ* agonist in MCF-7 and MDA-MB-231 human breast cancer cells. The importance of understanding the intracellular mechanism of action of anticancer agents is critical for optimizing therapeutic response. It is also clearly evident that use of *γ*-tocotrienol in combination with PPAR*γ* antagonist may have potential therapeutic value in treatment of breast cancer in women.
